# Unveiling blood biomarkers for neuronal hyperplasticity: Insights from AD molecular subtyping, a comprehensive review

**DOI:** 10.1002/alz.70475

**Published:** 2025-07-25

**Authors:** Niti Sharma, Danyeong Kim, Himadri Sharma, Moon Il Kim, Hyon Lee, Minju Kim, Nayoung Ryoo, Min Ju Kang, Jung‐Min Pyun, Young Ho Park, Jisun Ryu, Hyun Jung Oh, Hyun‐Sik Yang, Hang‐Rai Kim, Geon Ha Kim, Sangwon Han, YoungSoon Yang, Young Chul Youn, Charlotte Teunissen, Henrik Zetterberg, Philip Scheltens, Seong Soo A. An, Young‐Bum Kim, SangYun Kim, the Alzheimer's Disease All Markers (ADAM) Research Group

**Affiliations:** ^1^ Department of Bionano Technology Gachon Bionano Research Institute Gachon University Seongnam Republic of Korea; ^2^ Department of Neurology Veterans Medical Research Institute Veterans Health Service Medical Center Seoul Republic of Korea; ^3^ Department of Neurology Gachon University Gil Hospital Incheon Republic of Korea; ^4^ Department of Neurology Seoul National University College of Medicine & Seoul National University Bundang Hospital Seongnam Republic of Korea; ^5^ Department of Neurology Eunpyeong St. Mary's Hospital, The Catholic University of Korea Seoul Republic of Korea; ^6^ Department of Research and Development PeopleBio Inc Seongnam Republic of Korea; ^7^ Department of Neurology Brigham and Women’s Hospital/Harvard Medical School Boston USA; ^8^ Department of Neurology Dongguk University Ilsan Hospital Goyang Republic of Korea; ^9^ Department of Neurology Ewha Woman’s University Mokdong Hospital, Ewha Woman’s University College of Medicine Seoul Republic of Korea; ^10^ Department of Neurology Soonchunhyang University Seoul Hospital, Soonchunhyang University College of Medicine Seoul Republic of Korea; ^11^ Department of Neurology Soonchunhyang University Hospital Cheonan Republic of Korea; ^12^ Department of Neurology College of Medicine Chung‐Ang University Seoul Republic of Korea; ^13^ Neurochemistry Laboratory Department of Clinical Chemistry Vrije Universiteit Amsterdam Amsterdam Netherlands; ^14^ Clinical Neurochemistry Laboratory Sahlgrenska University Hospital Mölndal Sweden; ^15^ Department of Neurodegenerative Disease UCL Institute of Neurology London UK; ^16^ UK Dementia Research Institute at UCL London UK; ^17^ Hong Kong Center for Neurodegenerative Diseases Science Park Hong Kong; ^18^ Wisconsin Alzheimer's Disease Research Center University of Wisconsin–Madison, Health Sciences Learning Center University of Wisconsin School of Medicine and Public Health Madison Wisconsin USA; ^19^ Department of Psychiatry and Neurochemistry Sahlgrenska Academy at the University of Gothenburg Mölndal Sweden; ^20^ Department of Neurology & Alzheimer Center Amsterdam University Medical Center Amsterdam Netherlands; ^21^ Division of Endocrinology, Diabetes, and Metabolism Beth Israel Deaconess Medical Center and Harvard Medical School Boston USA

**Keywords:** Alzheimer's disease, blood biomarkers, neuronal hyperplasticity, personalized treatment

## Abstract

Alzheimer's disease (AD) is the most prevalent neurodegenerative disorder, predominantly affecting the aging population. Early detection through biomarkers is essential for early intervention. Recent sub‐classification of AD through extensive cerebrospinal fluid (CSF) proteomic analyses revealed distinct characteristics of each subtype, necessitating tailored therapeutic strategies. While CSF proteomics has identified potential biomarkers, the need for non‐invasive and cost‐effective substitutions highlights the importance of blood‐based biomarkers (BBMs). This review is a comprehensive review that categorizes potential BBMs based on neuronal hyperplasticity (subtype 1), underlining their role in refining subtype classification and enabling precision medicine. Early AD is often marked by cortical and hippocampal hyperactivity, followed by hypoactivity during later stages of neurodegeneration. While the exact mechanisms remain unclear, factors like Ca^2+^, glutamate, amyloid beta, tau, genetic factors, and impaired glial function play a role. Advancements in blood‐based diagnostics would improve detection, individual treatment strategies, and evaluation of therapeutic response, eventually reducing the burden of AD on health‐care systems.

**Highlights:**

- Alzheimer's disease (AD; subtype 1) exhibits neuronal hyperplasticity, mild cortical atrophy, and moderate microglial activation.
- The neuronal hyperplasticity subtype of AD is characterized by an upregulation of synaptic and plasticity‐related proteins, distinguishing it from other AD subtypes.
- Identifying biomarkers specific to neuronal hyperplasticity would enable real‐time monitoring of therapeutic responses, allowing for individualized therapy as opposed to a “one‐size‐fits‐all” strategy.
- The treatments based on neuronal hyperactivity reduction, restoration of synaptic plasticity, and anti‐inflammation/metabolic dysfunction would be useful in this AD subtype.
- Blood‐based biomarkers offer a cost‐effective and accessible alternative to cerebrospinal fluid and neuroimaging methods.

## 1 INTRODUCTION

Alzheimer's disease (AD) is the most common type of neurodegenerative disease (ND), characterized by abnormal accumulation of amyloid beta (Aβ) and tau protein in the brain. It is increasing globally due to an increase in the aging population, and currently, there is no proven way to prevent or cure AD. As per recent statistics, > 55 million people are affected, with AD and other dementias worldwide resulting in ≈ $68 billion burden on the medical system in 2024.^1^ Due to the distressing outcome of dementia on AD patients and health‐care structures, finding ways for better management and cure of AD is of top priority. The pathological hallmarks of AD (Aβ and tau neurofibrillary tangles [NFTs]) can be diagnosed in cerebrospinal fluid (CSF) and tau positron emission tomography (PET) with additional neuroimaging tools like single‐photon emission computed tomography (SPECT) and magnetic resonance imaging (MRI) for neurodegeneration. Recently developed PET tracers for amyloid and tau are now reference tests for AD neuropathology, supplementing the conventional methods. CSF biomarkers consist of total tau (t‐tau), phosphorylated tau (p‐tau_181_), and Aβ42. Numerous studies have consistently shown that the majority of patients diagnosed with AD have a common “AD biomarker profile” characterized by high p‐tau_181_ levels and low Aβ42 levels.^2^ MRI is useful for measuring brain atrophy patterns, whereas 2‐[^18^F] fluoro‐2‐deoxy‐D‐glucose (FDG) and amyloid PET assessed the glucose metabolism and Aβ burden in AD, respectively.^3^ An abnormal Core 1 (amyloid PET, biofluid marker Aβ42, p‐tau_181_, p‐tau_231_, p‐tau_217_) biomarker is sufficient for the diagnosis of AD to inform treatment decisions at any disease stage. Later, changing Core 2 biomarkers (biofluid marker MTBR‐tau_243_, other phosphorylated tau forms [e.g., p‐tau_205_], non‐phosphorylated mid‐region tau fragments, and tau PET) facilitates the prediction of disease progression.^4^

AD is a complex heterogeneous disease involving multiple pathways like synaptic function, neuroinflammation, and abnormal metabolism of Aβ, tau, and lipids. Given the heterogeneity in clinical symptoms, progression, and genetic factors across AD patients, personalizing treatment based on individual biomarkers, particularly in early diagnosis using blood‐based biomarkers (BBMs), is increasingly recognized as critical. The neuroimaging techniques (MRI and PET) are expensive, and CSF sampling requires a lumbar puncture (LP); the procedure is costly and requires specialized clinics. For the effective and timely treatment of AD, early detection of the disease is essential; hence, there is a serious need for diagnostic tools that potentially replace existing methods to swiftly and accurately recognize early symptoms of AD. Moreover, amyloid and tau plasma biomarkers demonstrated a significant association with the conventional biomarkers (amyloid PET, tau PET, CSF Aβ42, and CSF p‐tau_181_). In contrast, the correlation between plasma neurofilament light chain (NfL) and conventional biomarkers like MRI and FDG PET is not as strong. Additionally, plasma biomarkers showed high precision in identifying classic amyloid and tau biomarker positivity, with plasma Aβ42/p‐tau ratios displaying better performance.^5^ Recently, plasma p‐tau_217_ was reported to be strongly associated with AD pathology.^6^ Therefore, identifying BBMs in AD diagnostics is essential, as blood sampling is easy, cost effective, and possible at general clinics. Such a diagnosis could significantly reduce the wait times for treatment from several years to a few months.

Recent studies identified AD subtypes based on the large‐scale CSF proteomic analysis combining conventional markers, apolipoprotein E (*APOE*) variants, rare mutations, markers for vascular damage, patterns of cortical atrophy, and neuropathological characteristics^7^, ^8^ which provided significant insight into biological processes involved in AD. Each subtype also possesses unique clinical and biomarker characteristics, further emphasizing the value of personalized treatment approaches. The five molecular subtypes of AD identified are subtype 1: neuronal hyperplasticity, subtype 2: innate immune activation, subtype 3: RNA dysregulation, subtype 4: choroid plexus dysfunction, and subtype 5: BBB dysfunction.^8^ The subtypes had different clinical attributes, such as subtype 1 through 3 showing elevated t‐tau and p‐tau levels in CSF; subtype 1 had the longest average survival time, while subtype 3 had the shortest; subtypes 2 and 5 had the highest dementia progression rate, while subtype 4 had the lowest.^8^ The existence of individual biomarker signatures for each type further highlights the potential for exclusive therapeutic approaches based on the distinct molecular processes underlying each subtype.

In this review, we critically evaluated putative BBMs for neuronal hyperplasticity in AD (subtype 1) and their putative roles in streamlining AD subtyping and enhancing precision medicine in research into ND. Therapies targeting synaptic plasticity and neuronal resilience, such as neurotrophic factor modulators or synaptic enhancers, could prove to be more efficacious in subtype 1 patients. BBMs could also allow treatment efficacy to be tracked continuously, enabling the timely adjustment of therapeutic strategies based on how a person is responding.

## 2 MATERIALS AND METHODS

We searched three major databases (PubMed, Google Scholar, and Science Direct) with specific terms and inclusion/exclusion rules. Based on this approach, we aimed to cover the most relevant and high‐quality human studies available before February 2025. The literature search on PubMed used the following strategy: “plasma biomarker,” “blood biomarkers” AND “Alzheimer's disease” AND “cognitive change” AND “neuronal hyperplasticity.” We included original human studies reporting BBMs in the context of AD‐related cognitive change, hyperactivity, or molecular subtype 1. Studies were excluded if they were: (1) unrelated to AD; (2) animal‐only research; (3) reviews, conference abstracts, or editorials; (4) duplicate reports; or (5) informal publications (e.g., preprints, non–peer‐reviewed material). Although not a systematic review, we aimed to ensure methodological transparency and comprehensive coverage by applying clearly defined inclusion and exclusion criteria across the selected databases.

## 3 NEURONAL HYPERPLASTICITY IN AD

**FIGURE 1:**
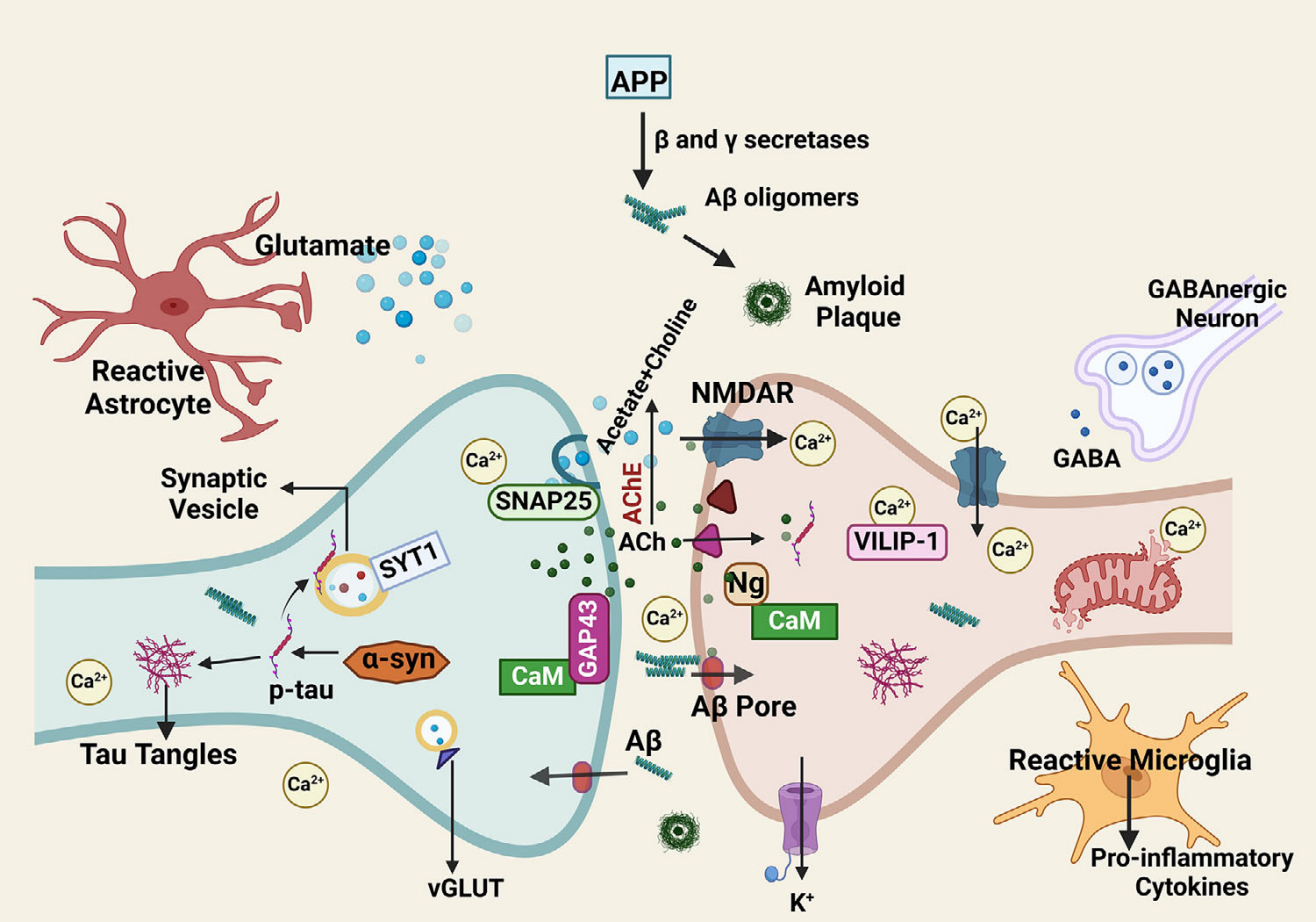
Events at the synapse of a hyperactive neuron in the AD brain. The elevated neuronal activity results in higher levels of Aβ. Ion channel pores (Aβ pores) can be formed in the plasma membrane by Aβ. Overaccumulation of Aβ instigates phosphorylation and secretion of tau, which in turn correlates with microglial dysfunction and reactive astrocytosis, culminating in synaptic dysfunction and neurodegeneration. Reactive astrocytes increase the release of glutamate. Elevated activation of calcium release from intracellular reservoirs in presynaptic and postsynaptic neurons leads to elevated cytosolic calcium concentrations. Enhanced glutamatergic signaling is caused by reduced astrocytic uptake or increased vGLUT expression. NMDAR is stimulated by increased glutamate release and reduced glutamate uptake. The release of pro‐inflammatory cytokines by activated microglia further enhances the excitability. Reactive astrocytes also increase neuronal excitability by reducing synaptic inhibition. Another factor contributing to AD hyperexcitability is the decline in firing frequency and quantity of inhibitory GABAergic neurons. Ng and GAP‐43 are CaM binding proteins present in the synapse. Other proteins like α‐syn, VILIP‐1, SYT‐1, and SNAP also affect synaptic functioning. AChE hydrolyzes Ach to acetate and choline. It also plays an important role in Aβ deposition in the brain. The Aβ–AChE complex is more neurotoxic than Aβ. Cleavage of amyloid precursor protein (APP) by BACE1 and γ‐secretase generates Aβ peptides, which subsequently form aggregates. Created with BioRender.com. Aβ, amyloid beta; α‐syn, α‐synuclein; Ach, acetylcholine; AChE, acetylcholinesterase; AD, Alzheimer's disease; BACE1, beta‐secretase 1; CaM, calmodulin; GAP‐43, growth‐associated protein 43; Ng, neurogranin; NMDAR, *N*‐methyl‐D‐aspartate receptor; p‐tau, phosphorylated tau; SNAP, synaptosome‐associated protein; SYT‐1, synaptotagmin 1; vGLUT, vesicular glutamate transporter; VILIP‐1, visinin‐like protein 1.

Neuronal hyperplasticity is unwarranted/atypical neuronal growth and synaptic remodeling. Such neurons actively express several proteins and growth factors related to cell proliferation, neurodevelopment, and plasticity. This was seen in subtype 1 of AD,^8^ which could be a brain compensatory mechanism to cope with cognitive disruptions caused by amyloid plaques and tau tangles by making new synaptic connections. Some studies have demonstrated increased neuronal activity in the cortical and hippocampal regions during early AD, transitioning to reduced activity as neurodegeneration advances.^9^ However, the specific reasons for alterations in neuronal excitability have not been completely identified. A dysfunction of inhibitory neurons has been linked to increased excitability in AD, where excessive glutamate release overstimulates neurons, leading to excitotoxicity. Decreased inhibitory tone in *APOE* ε4 mice has been linked to decreased sensitivity to GABAergic signals, fewer GABAergic interneurons, and reduced GABAergic signaling.^10^ New findings indicate that revising unusual neuronal activity in brain regions impacted in the initial stages of Aβ buildup not only decreases Aβ accumulation in that specific area but also stops the spread of Aβ‐related issues to other parts of the brain.^11^ Elevated neuronal activity results in higher levels of Aβ and triggers the release of tau, contributing to cognitive impairment.^12^ The synaptic origin for neuronal hyperactivity is the most accepted hypothesis,^13^, ^14^, ^15^ though the mechanism may differ, namely, increased soluble Aβ oligomers,^13^, ^14^ dysfunction in voltage‐gated sodium channel,^15^ and intracellular calcium store^16^ (Figure 1).

**FIGURE 2:**
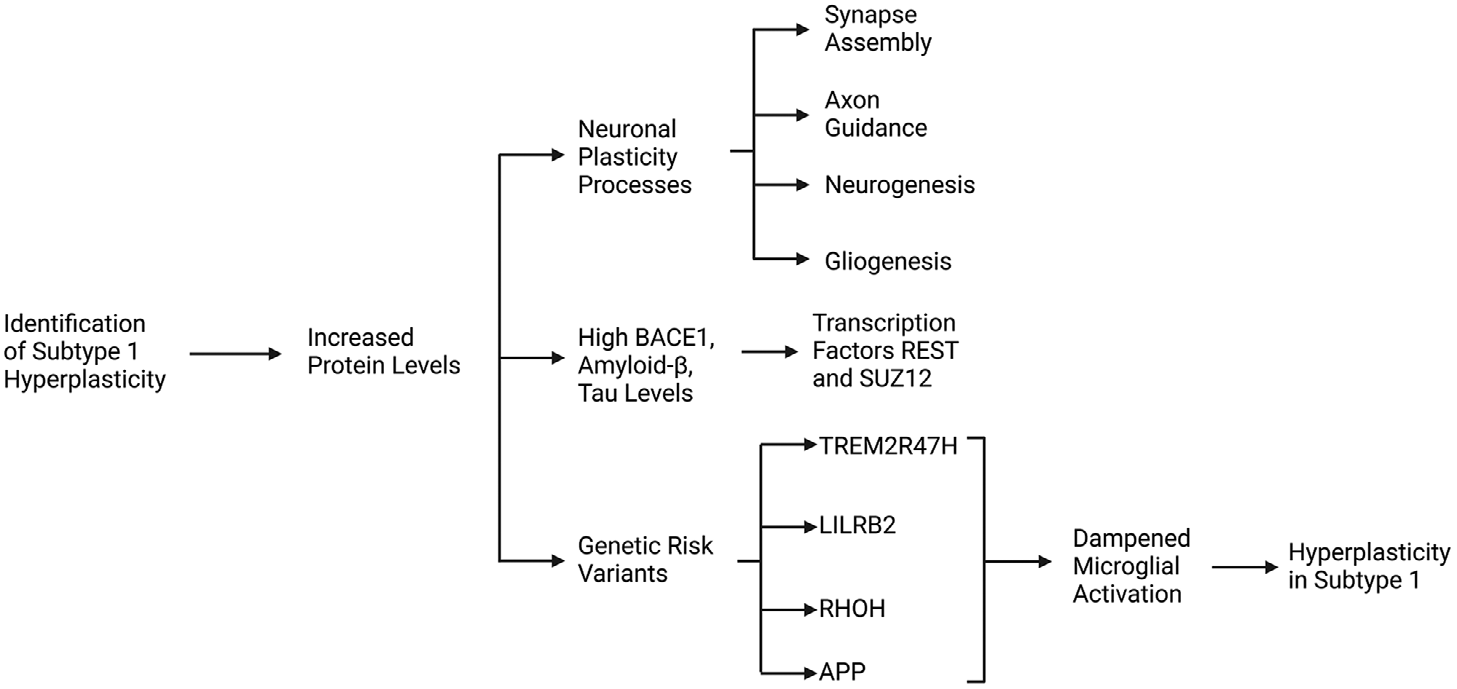
Important indicators and genetic risk factors of neuronal hyperplasticity in AD (subtype 1). AD, Alzheimer's disease; BACE1, beta‐secretase 1; REST, RE1 silencing transcription factor; SUZ12, polycomb repressive complex 2 subunit.

Subtype 1 exhibited increased CSF levels of protein related to neuronal plasticity (synapse formation, axon guidance, neurogenesis, and gliogenesis).^8^ This subtype exhibited milder brain atrophy and has the greatest percentage of proteins specific to neurons and glial cells compared to other AD subtypes. Important indicators include elevated levels of beta‐secretase 1 (BACE1), Aβ40, tau, and lysosomal protein (PLD3), along with genetic risk variations like triggering receptor expressed on myeloid cells 2 (*TREM2*) *R47H* mutation, leukocyte immunoglobulin‐like receptor subfamily B member 2 (*LILRB2*), Ras homolog family member H (*RHOH*), and amyloid beta precursor protein (*APP*), highlighting possible mechanisms behind this excessive growth reaction. Both RE1 silencing transcription factor (REST) and SUZ12 polycomb repressive complex 2 subunit (SUZ12) regulate APP and tau, which could be the reason for the simultaneous rise in Aβ production markers and tau levels.^17^ *TREM2 R47H* has been known to differentially affect the genes linked to synaptic transmission, neuronal signaling, synaptic vesicle traffic, and survival of neurons.^18^ It can decrease microglial activation,^19^ leading to difficulties in plaque consolidation and synapse pruning, which may cause axonal damage and increased synaptic plasticity as compensatory reactions^20^ (Figure 2). Levels of plasma soluble TREM2 (sTREM2) and CSF sTREM2 were noticeably elevated in the AD group compared to the mild cognitive impairment (MCI) and healthy control groups.^21^

The CSF levels of synaptic biomarkers increase with age and are linked to increased levels of CSF p‐tau and NfL. Previous research has suggested a two‐phase model for variations in cortical thickness in AD progression, in which cortical thickness might first rise in the early stages of the disease, possibly in response to inflammation or neuronal hypertrophy, and the cortical thickness declines as the disease progresses.^22^ Increased levels of CSF neurogranin (Ng) and growth‐associated protein 43 (GAP‐43) were found to be linked to elevated brain metabolism but reduced cortical thickness in AD‐affected brain areas, indicating they may be more closely linked to synaptic dysfunction than other markers of brain metabolic and structural changes.^23^ The CSF proteomic analysis revealed the enrichment of proteins responsible for synaptic plasticity, including the mitogen‐activated protein kinase/extracellular‐signal‐regulated kinase (MAPK/ERK) pathway, glucose metabolism, synaptic structure/function, and axonal development.^8^ PLD3 is accumulated in neuritic plaques and is involved not just in autophagy but also in inflammation^8^ in AD. It also controls the clearance of Aβ in both cell‐dependent and cell‐independent ways, which may play a role in the risk of developing AD.^24^ Generally, PLD3 expression is not noticeable under normal conditions but consistently increases in the activated response microglia (ARMs) state. Both transgene and APP knock‐in models demonstrated a 4‐fold rise in PLD3 levels in ARMs.^25^ PLD3 can be measured in serum and plasma using commercially available human enzyme‐linked immunosorbent assay (ELISA) kits, but until now, there is no literature suggesting its evaluation for AD.

Presynaptic dystrophic neurites that edge amyloid plaques are the areas of microtubule disruption, BACE1 elevation, and increased Aβ generation in AD,^26^ which describes elevated BACE1, Aβ, and PLD3 in this subtype. Two important glycolytic enzymes (aldolase A and pyruvate kinase) were found upregulated in the CSF of AD patients,^27^ indicating dysregulation of glucose metabolism. In clinics, blood tests for aldolase A and pyruvate kinase are being done to detect muscle damage and hemolytic anemia, respectively. However, no reference was available for their quantification in the blood for AD. In AD, most synaptic proteins are elevated in the CSF, but the literature on their detection in the blood is limited. This might be due to their relatively low concentration, less stability, or interfering blood proteins that hamper their detection.^28^

In Table 1, we discuss various potential BBMs for neuronal hyperplasticity available in the literature.

**TABLE 1:** Potential blood biomarkers for neuronal hyperplasticity.

Protein	^a^Gene	^a^Protein expression and localization	^a^Protein function	^a^Molecular function	Trend in AD	Assay	Refs.
Aβ42/40 ratio	*APP*	Cytoplasmic expression in the CNS and a few other tissues	Functions as a cell surface receptor and performs physiological functions on the surface of neurons relevant to neurite growth, neuronal adhesion, and axonogenesis	Heparin‐binding, Protease inhibitor, Serine protease inhibitor	Decrease (plasma, serum)	ELISA, SIMOA	^1^, ^2^, ^3^, ^4^, ^5^
Acetylcholinesterase	*AChE*	Membrane, intracellular	Hydrolyzes rapidly the acetylcholine neurotransmitter released into the synaptic cleft, allowing termination of the signal transduction at the neuromuscular junction. Role in neuronal apoptosis	Blood group antigen, hydrolase, serine esterase	Increase (plasma)	Colorimetric, ELISA	^6^, ^7^
β‐Secretase 1	*BACE1*	Granular cytoplasmic expression in several tissues	Responsible for the proteolytic processing of the amyloid precursor protein	Aspartyl protease, hydrolase, protease	Increase (plasma, serum)	ELISA, fluorescence	^4^, ^8^, ^9^, ^10^
Brain‐derived neurotrophic factor	*BDNF*	Cytoplasmic expression mainly in CNS	During development, promotes the survival and differentiation of selected neuronal populations of the peripheral and central nervous systems. Participates in axonal growth, pathfinding, and in the modulation of dendritic growth and morphology	Growth factor	Increase (plasma); decrease (serum)	ELISA Luminex assay	^11^, ^12^, ^13^, ^14^
Growth‐associated protein 43	*GAP‐43* (*B‐50, GAP‐43, PP46*)	Selective expression in the CNS and peripheral nerves	This protein is associated with nerve growth. It is a major component of the motile “growth cones” that form the tips of elongating axons. Plays a role in axonal and dendritic filopodia induction	Calmodulin‐binding, developmental protein	Decrease (NDEs)	ELISA	^15^
Matrix metalloproteinase‐9	*MMP‐9*	Selective nuclear and cytoplasmic expression in a subset of immune cells	Role in local proteolysis of the extracellular matrix and in leukocyte migration	Hydrolase, metalloprotease, protease	Increase (plasma)	ELISA	^16^, ^17^
Neurogranin	*NRGN* (RC3)	Cytoplasmic & nuclear expression in CNS	Acts as a “third messenger” substrate of PKC‐mediated molecular cascades during synaptic development and remodeling. Binds to calmodulin in the absence of calcium	Calmodulin‐binding	Decrease (NDEs)	ELISA	^15^, ^18^
Synaptotagmin 1	*SYT‐1* (*P65, SVP65, SYT*)	Selective expression in neutrophil in CNS	Calcium sensor that participates in triggering neurotransmitter release at the synapse	Differentiation	Decrease (NDEs)	ELISA	^15^
Synaptosome‐associated protein 25	*SNAP‐25* (*bA416N4.2, Dj1068F16.2, RIC‐4, RIC4, SEC9, SNAP, SNAP‐25*)	Distinct expression CNS	Involved in the molecular regulation of neurotransmitter release. May play an important role in the synaptic function of specific neuronal systems		Decrease (NDEs); increase (plasma)	ELISA SIMOA	^15^, ^19^, ^20^, ^21^, ^22^
β‐Synuclein	*SNCB*	Selective expression in neuropil and cells in the CNS	Non‐amyloid component of senile plaques found in AD. Could act as a regulator of SNCA aggregation process. Protects neurons from staurosporine and 6‐hydroxy dopamine (6OHDA)‐stimulated caspase activation in a p53/TP53‐dependent manner. Contributes to restoring the SNCA anti‐apoptotic function abolished by 6 OHDA		Increase (plasma, serum)	IP‐MS, MS, PET	^23^, ^24^, ^25^, ^26^
Triggering receptor expressed on myeloid cells 2	*TREM2*	Cytoplasmic and membranous expression in several tissues	Involved in microglial activation and phagocytosis of myelin debris after neuronal injury and of neuronal synapses during synapse elimination in the developing brain. Triggers activation of the immune responses in macrophages and dendritic cells	Receptor	Increase (plasma)	ELISA	^27^
Kallikrein‐related peptidase 8	*KLK‐8* (*HNP, neuropsin, ovasin, PRSS19, TADG14*)	Selective expression in squamous epithelium	Capable of degrading several proteins, also cleaves L1CAM in response to increased neural activity. Induces neurite outgrowth. Has a role in the formation and maturation of orphan and small synaptic boutons in the Schaffer‐collateral pathway, regulates Schaffer‐collateral LTP in the hippocampus, and is required for memory acquisition and synaptic plasticity	Hydrolase, protease, serine protease	Increase (serum)	ELISA	^28^, ^29^
Visinin‐like protein 1	*VSNL1* (*HLP3, HPCAL3, HUVISL1, VILIP, VILIP‐1*)	Not available	It is a neuronal calcium sensor protein. It is strongly expressed in granule cells of the cerebellum where it associates with membranes in a calcium‐dependent manner and modulates intracellular signaling pathways of the CNS by directly or indirectly regulating the activity of adenylyl cyclase	Calcium, metal‐binding	Increase (serum, blood)	ELISA SIMOA	^30^, ^31^

Abbreviations: Aβ, amyloid beta; AD, Alzheimer's disease; CNS, central nervous system; ELISA, enzyme‐linked immunosorbent assay; IP‐MS, immunoprecipitation mass spectrometry; L1CAM, L1 cell adhesion molecule; LTP, long‐term potentiation; MS, mass spectrometry; NDEs, neuronally derived extracellular vesicle; PET, positron emission tomography; PKC, protein kinase C; SIMOA, single molecule arrays.

^a^ Information obtained from The Human Protein Atlas.

### 3.1 Amyloid precursor protein

Proteolytic cleavage of amyloid precursor protein (APP) by beta and γ‐secretase results in the formation of Aβ40 and Aβ42 peptides, which have a significant role in AD. Aβ42 is the main component of Aβ plaques in AD brains, whereas Aβ40 is found in only a few plaques, indicating that the beginning stages of plaque formation involve only Aβ42.^29^ Aβ40 is the predominant type of amyloid peptide in CSF and plasma, being 10 to 20 times more common than Aβ42 in both healthy and diseased conditions. Researchers often analyze the Aβ42/Aβ40 ratio along with individual measurements of Aβ40 and Aβ42 to differentiate between patients with or without AD. Additionally, the Aβ42/Aβ40 ratio is especially valuable as a continuous indicator of the advancement of AD.

In AD pathology, Aβ peptide deposits can be detectable up to 20 to 30 years before the onset of clinical dementia and can differentiate AD‐type dementia from MCI.^30^ The concentration of Aβ peptides in blood is at least 100‐fold lower than in CSF; hence, its detection requires ultrasensitive immunoassays. Aβ42 is the component of senile plaques, while Aβ40 is present in CSF, and a decreased Aβ42/40 ratio is a strong biomarker of early AD. *APOE* genotypes affect individual Aβ40 and Aβ42 values, while the Mini‐Mental State Examination (MMSE) score is slightly associated with Aβ40 levels.^31^ In amnestic mild cognitive impairment (aMCI) and AD, significantly lower plasma Aβ42/40 was found compared to the control and matched the CSF Aβ42/40 profile. Also, plasma Aβ42/40 was inversely (*r*_s _= −0.464, *P* < 0.001) related to neocortical amyloid deposition.^32^ A recent study reported a significantly superior area under the curve (AUC) for plasma Aβ42/40 compared to other AD plasma biomarkers (p‐tau_181_, glial fibrillary acidic protein, and NfL), suggesting its ability to detect early amyloid accumulation.^33^ Usually, yearly and age‐dependent variation in the Aβ40/42 ratio is insignificant, but if the change is > 14.7%, other biomarkers also need to be examined.^34^

These results validate the efficacy of the plasma Aβ42/40 ratio in detecting early amyloid accumulation in AD, which is useful as a non‐invasive and low‐cost screening method for brain amyloidosis that can effectively distinguish between patients with positive and negative amyloid status. However, the small decrease in the plasma Aβ42/40 ratio (compared to the CSF Aβ42/40 ratio) in Aβ plaque pathology–positive people makes the test not very robust.^35^

### 3.2 Acetylcholinesterase

Acetylcholinesterase (AChE) is a key enzyme in cholinergic neurotransmission, which is consistently reduced in the AD brain. Even with the general reduction, AChE levels rise near Aβ∖ plaques, suggesting the involvement of AChE in Aβ fibril formation. Elevated AChE activity was specifically observed in the plasma from AD patients (22.40 ± 0.95 nmol/min/ml) compared to controls (18.71 ± 1.00 nmol/min/ml). This rise is linked to an increase in light isoforms (G1 and G2), which are elevated in the AD brain.^36^ Plasma AChE levels and their enzyme activity showed a negative correlation (AChE, *n* = 241, **P = 0.0015, *r* = −0.2035; enzymatic activity, *n* = 240, ***P = 0.0008, *r* = −0.2151) with the extent of Aβ accumulation.^37^ Hence, blood AchE has the potential to serve as a blood biomarker for predicting cerebral Aβ deposition in cognitively normal individuals.

### 3.3 Beta secretase 1

Beta secretase 1 (BACE1 is a β‐site APP‐cleaving enzyme 1, which is significantly increased in AD and MCI. Increased levels of BACE1 were reported in CSF in early AD compared to the healthy control.^38^ BACE1 plasma levels in AD were increased to 32%^39^ and 14.4%^40^ compared to non‐AD controls. The association of MMSE scores and BACE1 (sensitivity 71.4%; specificity 95%) was significantly better as a diagnostic tool compared to BACE1 (sensitivity 76.2%; specificity 65%).^40^ Shen et al. demonstrated the increased BACE1 levels in the plasma of AD and MCI patients (*V*_mean_ < 2 mFu/min/µg) compared to the control (*V*_mean_ > 2.6 mFu/min/µg).^41^ In addition, higher BACE1 was observed in MCI patients converted to probable AD, suggesting that plasma BACE1 can also detect AD progression. Lately, significantly higher serum levels of BACE1 (*P *< 0.001) have been reported in late‐onset AD (LOAD),^42^ MCI,^43^ and AD+AD‐MCI^44^ patients compared to the control. A strong connection of BACE1 with Aβ (measured in either CSF or through PET scan), t‐tau (in CSF), and cognitive function was also discovered.^45^ In addition, plasma long non‐coding RNA (LncRNA) BACE1 level in AD was significantly higher than that of healthy controls (*P*  =  0.006).^46^

### 3.4 Brain‐derived neurotrophic factor

Brain‐derived neurotrophic factor (BDNF) is a neurotrophin responsible for promoting the survival, growth, and differentiation of neurons and synaptic plasticity in the brain. It protects neurons against tau‐related degeneration despite the association between BDNF depletion and Aβ buildup, tau phosphorylation, neuroinflammation, and neuronal apoptosis.^47^ Various studies have suggested that blood BDNF levels may serve as a biomarker for the diagnosis and monitoring of AD. BDNF levels were significantly (*P* < 0.001) higher in AD (2545.3 pg/ml) than in controls (1503.8 pg/ml).^48^ A significant increase in plasma BDNF was also seen in MCI (3.19 ± 0.37 pg/ml) compared to in the healthy control (2.34 ± 0.51 pg/ml, *P *< 0.001). It also showed a strong ability to differentiate between MCI and healthy controls with high accuracy (β = 0.47, 95% confidence interval [CI] = 0.32 to 0.62, *P* < 0.001, *R* ^2 ^= 0.44).^49^ Similar results were reported by Qian et al., and additionally, plasma BDNF levels were significantly (*P *< 0.001) correlated with Clinical Dementia Rating (CDR), MMSE, and clinical diagnosis. Initially, BDNF decreased and later increased with cognitive impairment in the *APOE* ε4‐negative group (*P* < 0.05).^50^ The rise in BDNF could indicate a compensatory response to early neurodegeneration and appears to be associated with inflammation. As the disease's severity advances, these compensatory mechanisms might start to dysfunction, dropping BDNF levels in the peripheral blood.

BDNF levels were also evaluated in the serum. The connection between serum BDNF and AD progression has been associated with the pace of cognitive decline. Reduced serum BDNF levels are specifically linked to rapid cognitive decline in AD, rather than gradual cognitive decline.^51^ The relationship also exists between serum pro‐BDNF levels and hippocampal pro‐BDNF levels, which are associated with hippocampal p‐tau expressions.^52^ A combination of proBDNF and mature BDNF (Mbdnf)/proBDNF was proposed to improve diagnostic efficiency.^53^ The AD‐MCI group showed significantly (*P* = 0.037) lower serum BDNF levels compared to the healthy group. Additionally, a positive correlation was observed between blood BDNF and CSF Aβ42 levels (*r* = 0.49, *P* = 0.005). A decline in serum BDNF levels was linked to a decrease in CSF Aβ42 levels, which may be related to medial temporal lobe atrophy caused by the progression of tau pathology.^54^ Recently, the serum plasminogen activator inhibitor‐1 (PAI‐1)/BDNF ratio was suggested as a marker for AD. The elevated serum levels of PAI‐1 in AD retard BDNF production. Hence this ratio can be used to predict AD and it is also inversely related to MMSE scores.^55^ BDNF can be released from the platelets; therefore, plasma should be processed to estimate BDNF levels. The platelet‐rich plasma (PRP) had significantly (*P* < 0.001) greater BDNF levels compared to the platelet‐poor plasma (PPP). Moreover, PPP BDNF was found to be positively correlated (β = 0.645, *P *= 0.008) with p‐tau_181_, a known AD biomarker.^56^ Janel et al. suggested a combined assessment of plasma dual specificity tyrosine phosphorylation‐regulated kinase 1A (DYRK1A) and homocysteine with BDNF to increase accuracy (93.3%), sensitivity (95.2%), and specificity (88.9%) of the detection.^57^

### 3.5 Growth associated protein ‐43

Growth associated protein ‐43 (GAP‐43 is a calmodulin (CaM)‐binding protein exclusively present in the excitatory neurons in the nervous system, found upregulated in conditions like epilepsy. It is also known as neuronal plasticity protein owing to its high expression in neuronal growth cones during axonal regeneration and involvement in neurotransmitter release and long‐term potentiation (LTP). The overexpressed GAP‐43 boosts sprouting after injury and creates new synapses.^58^ The binding of CaM to GAP‐43 inhibits phosphorylation of GAP‐43 at Ser41 by protein kinase C (PKC), preventing activation of GAP‐43 in response to transient changes in second messenger.^59^ CSF levels of GAP‐43 were notably higher in AD patients compared to controls and other NDs and showed a positive connection with the extent of deposition of NFT and Aβ plaques in the hippocampus, amygdala, and cortex. However, no link was found between GAP‐43 and α‐synuclein (α‐syn) or TAR DNA‐binding protein 43 (TDP‐43) pathology.^60^ Recently, a connection between the potential synaptic changes in faster Aβ‐related tau spreading and higher CSF GAP‐43 levels in AD patients has been reported.^61^

GAP‐43 has a role in neurotransmitter release and synaptic activity, and its CSF levels have been reported to increase in AD.^62^ Another study using blood neuronally derived exosomes (NDEs) registered a decrease in NDE GAP‐43 levels in AD (1926 ± 509 pg/ml) and aMCI (2325 ± 606 pg/ml) compared to the control (2722 ± 664 pg/ml).^63^ Besides GAP‐43, myristoylated alanine‐rich C‐kinase substrate (MARCKS), and brain acid soluble protein 1 (BASP1) are also highly expressed in the axon endings and participate in signal transmission and cytoskeleton organization.^64^ An elevated level of pSer46‐MARCKS was reported in the serum and CSF of patients with early‐stage frontotemporal lobar degeneration (FTLD),^65^ and an increased expression of BASP1 was reported in the AD mice brain.^66^ However, the literature about the expression in the blood of AD patients is unavailable for both these proteins. As the presynaptic marker, GAP‐43 is linked to the pathological features of AD, it can be used as a precise and specialized biomarker for clinical studies.

### 3.6 Matrix metalloproteinase‐9

Matrix metalloproteinase‐9 (MMP‐9) is a zinc‐dependent category of extracellular endopeptidases expressed by neurons and glial cells. It is essential for synaptic plasticity, memory and learning, tissue remodeling, and axon regeneration.^67^ MMP‐9 is required for converting proBDNF into Mbdnf, critical for cell survival and synaptic adaptability. Research has indicated elevated plasma MMP‐9 concentrations in MCI and AD.^68^, ^69^ The findings revealed that plasma BDNF levels had a significant positive correlation (β [95% CI] = 0.454 [0.403–0.705], *P* < 0.001) with plasma MMP‐9 levels.^70^

### 3.7 Neurogranin

Neurogranin (Ng), a CaM‐binding post‐synaptic protein, is expressed in the neurons of the hippocampus and cerebral cortex and participates in the regulation of synaptic plasticity. It has been recognized as an early biomarker of CNS infection and synaptic dysfunction. A link among elevated Ng levels in CSF, brain atrophy, amyloid LOAD, and cognitive decline has also been well documented in AD.^71^ After the advent of the NDE detection technique in plasma, Jia et al. documented a reduced level of Ng in AD (254 ± 69 pg/ml) compared to the control (2099 ± 540 pg/ml) in NDEs, which was also significantly associated with cognitive decline (*R* ^2^ = 0.60; *P *< 0.0001).^63^ Ng concentration in blood plasma exosomes was lower in AD and MCI patients compared to healthy controls, and even lower in AD and MCI‐AD patients compared to stable MCI (sMCI) patients (*P* < 0.001).^72^ The Ng levels increase in CSF, but in NDEs, they displayed the contrary trend, which could be due to the transport of Ng from plasma into the CSF.^73^ These results demonstrate that blood exosome Ng can also serve as a cognitive biomarker for AD and MCI‐AD, with additional research required to determine the precise Ng values for diagnosis in various stages of AD.

### 3.8 Synaptotagmin 1

Synaptotagmin 1 (SYT‐1) is a calcium sensor with a role in the synchronous release of neurotransmitters in the hippocampus. CSF SYT‐1 showed potential as a biomarker for tracking synaptic dysfunction and degeneration in AD. Elevated levels of CSF SYT‐1 were found in patients with AD‐related dementia (*P* ≤ 0.0001) and AD‐related MCI (*P* < 0.001).^74^

Using NDEs, a lower concentration of SYT‐1 was detected in AD (312 ± 81 pg/mL) compared to healthy controls (586 ± 153 pg/mL).^63^ The study promoted exosomal GAP‐43, Ng, and SYT‐1 as effective biomarkers that could be used in combination to predict AD at the asymptomatic stage.

### 3.9 Synaptosome‐associated protein 25

Synaptosome‐associated protein 25 (SNAP‐25) is a part of the soluble *N*‐ethylmaleimide‐sensitive‐factor attachment protein receptor (SNARE) protein complex, which is involved in the exocytotic release of neurotransmitters during synaptic transmission. Recently, non‐SNARE roles and postsynaptic functions have also been identified for SNAP‐25.^75^ SNAP‐25 is increased in the CSF of AD patients and serves as a biomarker for synaptic activity.^76^ Decreased levels of SNAP‐25 were detected in serum NDEs, which correlated with cognitive status (*r*  =  0.465; *P*  =  0.01). NDEs SNP‐25 could differentiate between AD and control (AUC  =  0.826, sensitivity  =  87.5%, specificity  =  70.6%).^63^, ^77^ With the development of a novel ultra‐sensitive immunoassay called single molecular arrays (Simoa), it was possible to detect SNAP‐25 in the plasma of AD patients. Nilsson et al. found a strong correlation between immunoprecipitation‐mass spectrometry (IP‐MS) and Simoa methods in detecting AD.^78^ The elevated plasma level of SNAP‐25 in AD (0.81 pg/mL vs. 0.6 pg/mL) had a significant correlation with MMSE (β = −6.69, *P* = 0.001), cortical atrophy (β = −0.05, *P* = 0.009), and amyloid centiloid (*P* = 0.016, rho = 0.48).^79^, ^80^ Hence, SNAP‐25 quantification in blood could provide an easily available measure of synaptic integrity in AD.

### 3.10 β‐synuclein

β‐synuclein (SNCB) is a presynaptic protein predominantly expressed in the brain. SNCB is altered in preclinical AD much before p‐tau_181_, suggesting its use as an early AD biomarker. Furthermore, SNCB levels are altered comparatively more in amyloidopathies; their elevated levels in CSF act as an early biomarker of AD. Lately, immunodetection techniques have increased the sensitivity of SNCB for effective quantification in blood.^81^ Serum SNCB increased significantly (*P* < 0.001) in demented‐AD patients compared to MCI‐AD patients versus control. The level of SNCB in the blood was found to be associated with amyloid PET positivity in various regions and hence showed distinguishing characteristics for Aβ PET–positive and negative individuals.^82^ Moreover, it did not correlate with the MMSE score.^81^ The serum SNCB increased significantly in AD (11.7 pg/mL vs. 9.4 pg/mL) and was strongly associated with regional brain atrophy regions observed by MRI. ^83^ Several other studies correlated blood SNCB levels with brain atrophy, cognitive impairment, and amyloid PET, signifying its connection with AD severity.^82^, ^83^, ^84^ Recently, the Simoa technique was developed to quantify SNCB in blood for prion disease but has not been studied in the AD scenario.^85^ The above research findings proved the eligibility of SNCB as a candidate blood marker for early synaptic degeneration that might be useful in clinical AD trials.

### 3.11 Kallikrein‐related peptidase 8

Kallikrein‐related peptidase 8 (KLK‐8; neuropsin), a serine protease important for neuroplasticity and memory acquisition, is expressed chiefly in the hippocampus. It regulates cell adhesion and extracellular matrix proteins for synapse remodeling. KLK‐8 specifically modifies the L1 cell adhesion molecule (L1CAM) in the presynaptic region in the hippocampus of mice.^86^ This modification disrupts synaptic integrity and functionality, resulting in the synaptic impairments seen in neurodegenerative disorders.^86^ Studies suggested that CSF and blood KLK‐8 can be potential biomarkers for detecting early AD and aMCI.^28^, ^87^ MCI patients had the highest blood KLK‐8 levels (1139.3 pg/mL), compared to AD (453.9 pg/mL) and controls (227 pg/mL), indicating blood KLK‐8 as a stronger discriminator for MCI (AUC = 0.94) than AD (AUC = 0.83)^87^ and is not associated with non‐amnestic MCI (naMCI).^88^ As far as diagnostic accuracy is concerned, CSF KLK‐8 equaled or surpassed that of CSF Aβ42, t‐tau, and p‐tau for both MCI and AD and was even superior to CSF Aβ42 in the case of MCI. Blood KLK‐8 was just as effective at distinguishing MCI but slightly less effective for AD compared to the core CSF biomarkers mentioned. Additionally, no significant correlations were observed between sex and *APOE* genotype in CSF or blood KLK‐8 values.^87^ This suggests that blood KLK‐8 might serve as a biomarker in the early detection of AD. However, there are sex‐specific variations in the relationship between KLK‐8 and cognitive decline during the initial stages of AD, which might result from varying levels of sex hormones.^89^ Thus, to precisely comprehend KLK‐8 blood levels, sex‐specific analyses are essential.

### 3.12 Visinin‐like proteins

Visinin‐like protein 1 (VILIP‐1), a neuronal calcium sensor protein, regulates synaptic plasticity and cognition by signaling cyclic nucleotides, promoting dendritic growth, modulating the nicotinergic activity, and indicating neuronal cell death. VILIP‐1 in blood and CSF is an emergent marker for early AD and other NDs. *APOE* ε4 carriers had a noteworthy rise in CSF VILIP‐1 levels, which showed a positive correlation with tau and p‐tau levels.^90^ CSF levels of VILIP‐1 and VILIP‐1/Aβ42 showed comparable predictive abilities for total cognitive decline as tau and tau/Aβ42.^91,^ ^92^ Serum VILIP‐1 is considered a better biomarker for epilepsy and seizure‐induced neuronal injury. VILIP‐1 mRNA expression has been associated with NFTs and MMSE scores of AD patients.^93^ Also, CSF levels of VILIP‐1 are correlated with tau and the *APOE* genotype in AD.^94^ Chen et al. noticed elevated VILIP‐1 in the blood of AD patients (9.0 ± 2.9 ng/L) compared to the control (3.3 ± 1.7 ng/L), and its levels were negatively (*r* = −0.463; *P*<0.01) and positively (*r* = 0.417; *P* = 0.01) correlated with MMSE score and age, respectively.^95^ Increased VILIP‐1 levels were recently detected in serum (*P*<0.01) and CSF (*P*<0.0001) compared to controls. However, a weak correlation was observed between CSF and serum VILIP‐1 levels (*r *= 0.32; *P *< 0.0001).^96^ Because VILIP‐1 is involved in regulating synaptic plasticity, it could serve as an indicator of synaptic dysfunction in AD.

## 4 PATHOLOGICAL VERSUS COMPENSATORY HYPERACTIVITY IN AD SUBTYPE 1

While slight increased neural activity may have initially served as an adaptive response to early neurodegeneration, growing evidence suggested that the hyperactivity observed in subtype 1 reflected pathological overactivation rather than compensation. In a CSF proteomic study, the upregulation of synaptic and glutamatergic signaling proteins was reported in subtype 1, which aligned with excessive excitatory activity rather than healthy synaptic plasticity.^8^ Hyperactivity of hippocampal neurons led to Aβ deposition, signifying that hyperactivity is one of the earliest dysfunctions in the pathophysiological cascade of AD.^97^ In a mouse model of AD, soluble Aβ oligomers triggered neuronal hyperactivity, disrupted the neuronal network synaptic excitation/inhibition (E/I) balance, and contributed to cognitive decline, indicating a pathological condition.^98^ The mechanisms associated with AD amyloid pathology established that variations in neural activity controlled Aβ levels in the interstitial fluid.^99^ Furthermore, reducing this hyperactivity in the hippocampal cornu ammonis 3/dentate gyrus (CA3/DG) using low‐dose levetiracetam improved memory, supporting the notion that the overactivation is a pathogenic process.^100^ Medications that increased GABAergic inhibition, like pentobarbital, improved memory deficits in these models, suggesting that the hyperactivity was detrimental rather than helpful.^101^

In addition to individuals with aMCI, *APOE* ε4 carriers and those with familial AD mutations also exhibited hippocampal hyperactivity. Initially believed to be a compensatory response, several functional MRI (fMRI) studies had shown increased hippocampus activation in these groups,^102^, ^103^ correlating with cognitive decline rather than compensatory mechanisms. According to Dickerson et al.,^104^ aMCI individuals who exhibited greater hippocampus activation on fMRI during memory tasks were likely to undergo a more rapid cognitive decline over time. Other research indicated that this type of hyperactivity, particularly in the CA3/DG, was associated with worse memory function, suggesting a pathological condition in the brain.^105^ In a similar study, magnetoencephalography (MEG) recordings of brain electrical activity demonstrated that aberrant slowing in hippocampus regions corresponded with cognitive decline in early AD.^106^ All of these results lent credence to the theory that the hyperactivity observed in subtype 1 AD is a pathological process that affects memory rather than being adaptive.

Collectively, these findings suggested that the sustained, spatially localized hyperactivity observed in subtype 1 is not a transient, compensatory adaptation, but rather a disease‐driving mechanism that could be targeted therapeutically.

## 5 CLINICAL AND NEUROIMAGING RELEVANCE OF BBMS LINKED TO NEURONAL HYPERPLASTICITY IN AD

Current literature did not provide sufficient evidence directly linking the plasma levels of most BBMs mentioned in this review to neuroimaging or structural brain changes in AD. The available information^32^, ^54^, ^79^, ^107^, ^108^, ^109^, ^110^, ^111^, ^112^, ^113^, ^114^, ^115^, ^116^, ^117^, ^118^, ^119^, ^120^, ^121^ is presented in Table 2. To clarify the translational potential of the reviewed biomarkers, we overviewed their levels of validation and use in clinical trials in Table 3. Biomarkers, like the plasma Aβ42/40 ratio, have established cutoff values and are already being used in screening or enrichment in treatment trials.^115^, ^122^, ^123^, ^124^ Others, including BDNF, Ng, and SNCB, are at an earlier stage of clinical validation but showed a clear association with neuronal hyperplasticity (subtype 1) and synaptic function.^44^, ^45^, ^48^, ^63^, ^84^, ^87^, ^121^, ^125^

**TABLE 2:** Correlation between BBMs and neuroimaging markers in AD.

Biomarker	Neuroimaging/ structural correlation	Key findings	Refs.
**Aβ42/40 ratio**	Amyloid PET Tau PET FDG PET PiB PET PET SUVR SBM	Lower plasma Aβ42/40 ratios are significantly associated with higher cerebral amyloid and tau deposition, neocortical amyloid deposition, cortical thickness, hippocampal atrophy, and hippocampal‐dependent memory loss. Also correlated with age, *APOE* ε4 status, and brain amyloidosis.	^32^, ^33^, ^34^, ^35^, ^36^, ^37^
***AChE***	Amyloid PET	Increased plasma AChE activity correlates with higher amyloid plaque burden across multiple cortical regions, suggesting a link between peripheral cholinergic activity and central amyloid pathology.	^38^
BACE1	FDG PET, voxel‐based morphometry, PET	Elevated plasma BACE1 levels are associated with cerebral accumulation of Aβ, cognitive decline, atrophy in the basal forebrain and hippocampus, as well as decreased glucose metabolism in cognitively healthy individuals at risk for AD. BACE1 variant and *APOE* ε4 interaction have a role in brain Aβ deposition.	^39^, ^40^, ^41^, ^42^
BDNF	Amyloid PET, MRI	Lower plasma BDNF levels correlate with increased amyloid deposition and greater medial temporal lobe atrophy, indicating its potential as a biomarker for early AD detection.	^13^, ^43^
MMP‐9	MRI, ADNI	Accelerated hippocampal atrophy and cognitive decline. Faster memory loss in females.	^44^, ^45^
SNAP‐25	Amyloid PET, MMSE score	Elevated plasma SNAP‐25 levels in cognitively impaired individuals and amyloid and tau‐positive individuals	^21^
TREM2	Tau PET Florbetapir PET	Elevated plasma soluble TREM2 levels are associated with increased white matter lesions and tau pathology, independent of amyloid burden.	^46^, ^47^

Abbreviations. Aβ, amyloid beta; AChE, acetylcholinesterase; AD, Alzheimer's disease; ADNI, Alzheimer's Disease Neuroimaging Initiative; *APOE*, apolipoprotein E; BACE1, beta‐secretase 1; BBM, blood‐based biomarker; BDNF, brain‐derived neurotrophic factor; FDG PET, ^18^F‐fluorodeoxyglucose positron emission tomography; MMP‐9, matrix metalloproteinase‐9; MMSE, Mini‐Mental State Examination; MRI, magnetic resonance imaging; PET, positron emission tomography; PiB, Pittsburgh compound B; SBM, surface‐based morphometry; SNAP‐25, synaptosome‐associated protein 25; SUVR, standardized uptake value ratio; TREM2, triggering receptor expressed on myeloid cells 2.

**TABLE 3:** Status of BBMs in clinical trials.

Protein	Validation status	Clinical trial cut‐off values	In clinical trials	Refs.
**Aβ42/40 ratio**	✓	Plasma Aβ42/40 ratio ≥0.170: lower risk; Aβ42/40 ratio of 0.150 to 0.169: intermediate risk; Aβ42/40 ratio < 0.150: higher risk of AD Plasma Aβ42/Aβ40 (<0.1218)	NCT05026866 trial (TRAILBLAZER‐ALZ 3); TRAILBLAZER‐ALZ studies	^34^, ^48^, ^49^, ^50^
BACE1	○	20.7Ku/L (70%–80% specificity and sensitivity) in serum	Observational cohorts	^4^, ^51^, ^52^
		11.04 Ku/L (AUC 0.991) in serum		
		27 ± 7.4 pg/mL (plasma)		
BDNF	○	1497.4–4153.4 pg/mL (plasma) AD (1.16; 0.13–21.34); MCI (0.68; 0.02–19.14)	Observational cohorts	^12^, ^53^
GAP‐43	○	AUC value 0.83	Two‐stage‐sectional study	^15^
KLK‐8	○	≥ 397 pg/mL (for AD) and ≥ 1121 pg/mL (for MCI)	Observational cohorts	^54^
MMP‐9	○	AUC value 0.78	Observational cohorts	^55^
**Neurogranin**	○	Results awaited	ChiCTR2000029055	^56^
	○	AUC value 1.0	Two‐stage‐sectional study	^15^
SNAP‐25	○	AUC value 0.75	Two‐stage‐sectional study	^15^
SNCB	○	AUC value 0.77	Observational cohorts	^57^
SYT‐1	○	AUC value 0.95	Two‐stage‐sectional study	^15^
TREM2	○	≥ 460 pg/mL	Neurology database of Ruijin Hospital for Alzheimer's Disease (NRHAD) cohort	^47^

*Note*: ✓, Accepted (validated) use; ○, Potential use, supportive data available.

Abbreviations: Aβ, amyloid beta; AD, Alzheimer's disease; AUC, area under the curve; BACE1, beta‐secretase 1; BBM, blood‐based biomarker; BDNF, brain‐derived neurotrophic factor; GAP‐43, growth‐associated protein 43; KLK‐8, kallikrein‐related peptidase 8; MCI, mild cognitive impairment; MMP‐9, matrix metalloproteinase‐9; SNAP‐25, synaptosome‐associated protein 25; SNCB, β‐synuclein; TREM2, triggering receptor expressed on myeloid cells 2.

## 6 CONCLUSION AND FUTURE DIRECTIONS

Recent proteomics studies of CSF have revealed potential molecular signatures of neuronal hyperplasticity in AD (subtype 1), including mild cortical atrophy, moderate microglial activation, increased synaptic proteins, neurotrophic factors, and plasticity‐related kinases. These findings in the form of blood‐based diagnostics would assist in the early diagnosis and monitoring of disease progression. Plasma biomarkers such as Ng and BDNF have already established links with synaptic resilience and cognitive function in AD. Also, novel blood‐based indicators of axonal integrity could further enhance subtype 1 specificity. Consequently, the treatments in this specific AD subtype should focus on neuronal hyperactivity reduction, restoration of synaptic plasticity, and anti‐inflammatory/metabolic dysfunction. Monoclonal antibodies (donanemab, lecanemab, and aducanumab) promote the removal of amyloid plaques, diminishing hyperplasticity caused by amyloid aggregation. Cholinesterase inhibitors (donepezil, galantamine, and rivastigmine) enhance the neurotransmitter acetylcholine (Ach), supporting communication between cells in the brain. Memantine can be used to prevent calcium overload, protect against excitotoxicity, and preserve normal synaptic plasticity by serving as an antagonist at the *N*‐methyl‐D‐aspartate receptor (NMDAR). In addition, TREM2 activators would help prevent excessive synaptic remodeling and reduce amyloid‐driven hyperplasticity by enhancing microglial phagocytosis and Aβ clearance. Recently, a small molecule agonist (VG3927: Vigil Neuroscience) has demonstrated promising results in Phase 1 clinical trials.

Importantly, emerging evidence suggested that subtype 1 might have responded distinctively to strategies targeting hyperexcitability. For example, low‐dose levetiracetam, an anti‐epileptic agent, was shown to reduce hippocampal hyperactivity and to improve memory performance in individuals with aMCI—a clinical phenotype potentially aligned with AD subtype 1.^126^ Tijms et al.^8^ also suggested that subtype 1 is biologically enriched in pathways linked to synaptic activity and glutamate signaling, indicating that it could have been particularly receptive to treatments that targeted glutamatergic function, such as NMDAR antagonists.

Though existing anti‐amyloid treatments like lecanemab and donanemab are not yet personalized to specific molecular subtypes, a post hoc analysis evaluating treatment response by proteomic subtype (particularly those characterized by hyperactivity) might provide insights into subtype‐specific efficacy. Thus, designing clinical trials that consider these biomarker‐defined subtypes would be a key step toward more personalized and effective treatment approaches in AD. In hyperplasticity‐driven AD subtypes, these results offered credibility to the viability of multimodal approaches to improve diagnostic accuracy and track treatment outcomes. Further studies integrating blood biomarkers, imaging phenotypes, and clinical outcomes were essential to establish clinically actionable subtype classifications, optimize therapeutic strategies, and ultimately improve patient stratification in AD trials.

## CONFLICT OF INTEREST STATEMENT

H.Z. has served on scientific advisory boards and/or as a consultant for Abbvie, Acumen, Alector, Alzinova, ALZPath, Amylyx, Annexon, Apellis, Artery Therapeutics, AZTherapies, Cognito Therapeutics, CogRx, Denali, Eisai, LabCorp, Merry Life, Nervgen, Novo Nordisk, Optoceutics, Passage Bio, Pinteon Therapeutics, Prothena, Red Abbey Labs, reMYND, Roche, Samumed, Siemens Healthineers, Triplet Therapeutics, and Wave; has given lectures in symposia sponsored by Alzecure, Biogen, Cellectricon, Fujirebio, Lilly, Novo Nordisk, and Roche; and is a co‐founder of Brain Biomarker Solutions in Gothenburg AB (BBS), which is a part of the GU Ventures Incubator Program (outside submitted work). The rest of the authors have nothing to disclose. Author disclosures are available in the supporting information.

## Supporting information



Supporting information
